# Ohio State Dental Society

**Published:** 1883-12

**Authors:** 


W. D. MILLER,

*Secretary.*

---

### OHIO STATE DENTAL SOCIETY.

The eighteenth annual meeting of this society was held in the city of Columbus, commencing Wednesday, Oct. 31st, and continuing three days. The attendance, for various reasons, was less than usual, but the sessions were interesting and spirited.

The officers were as follows:

President, Dr. J. W. Lyder, of Akron; Vice-President, Dr. A. Berry, of Cincinnati; Secretary, Dr. J. H. Warner, of Columbus; Treasurer, Dr. G. W. Keeley, of Oxford.

Very little, save routine business, was accomplished at the morning session, and the society adjourned early. The State Board of Examiners met at noon, and two applicants for admission to practice were examined.

At the afternoon session, Dr. H. A. Smith suggested that the first two subjects "Etiology of Dental Caries" and "Is Dental Decay Unitary or Multiple," be discussed together. This was agreed to.

*Dr. J. Taft*—Said that to his apprehension the cause of the decay of teeth is more general than special. He enumerated and described a number of the causes leading to the disintegration of the organic substances making up the teeth, saying that in the formation of the teeth the organic portions are formed first, and then the inorganic, and that in their decay the inorganic matter goes first.

*Dr. H. A. Smith*—Spoke of the distinction between caries and what is called chemical abrasion. He recommended the use of nitrate of silver in the latter. He compared the different theories of dental decay as held at the present day. He presented the views of Dr. W. D. Miller, of Berlin, as opposed to those of Dr. Watt, and held to the belief that bacteria have something to do with the decay of teeth. He was of the opinion that Dr. Watt's hypothesis was not satisfactory. Investigators had not recognized the presence of the three acids, lactic, sulphuric, and nitric, save very rarely.

*Dr. A. Berry*—Holds that decay is unitary.

*Dr. F. H. Rehwinkle*—Said that the believers of the theories of chemical and bacterian causes of decay, seem inclined to come together in their views. Dr. Watt has not been correctly understood. When he speaks of an acid condition, he means the manufacture of a strong acid in the cavity of decay, which may be very destructive. Experiments made out of the mouth could not be conducted under the same conditions as those in the mouth.

*Dr. Smith*—Said that oxy-chloride of zinc would exercise a preservative influence on tooth tissue. With oxy-phosphate, the same result might be obtained by its imperviousness.

*Dr. J. A. Robinson*—Read a paper entitled "Dental Protection; Leaves from my Experience." It was principally devoted to the cleaning of the teeth. He referred to the universality of law, and said that when teeth decayed there was a reason for it. He drew a number of parallels between selected cases from his practice, in which all the conditions seemed alike, save that in one the teeth were regularly and faithfully cleaned, while in the other they were neglected. He detailed the varying conditions of the mouth and of the work done at the end of different periods of time, the results markedly proving that very much of decay of the teeth, as well as the failure of good fillings, is due to neglect and uncleanliness upon the part of the patient.

*Dr. Rehwinkle*—The profession is now all torn up over the remedies proposed. We are ever turning to new ones, which we are continually accepting on somebody's recommendation. Carbolic acid is now placed low down in the list of antiseptics. There is much of enthusiasm over eucalyptus, but its very disagreeable odor is exceedingly objectionable.

*Dr. H. A. Smith.*—Do you not recognize dental decay as a pathological condition?

*Dr. Rehwinkle.*—Well, if it be not so at its inception, it is very apt to soon become so.

*Dr. C. R. Butler*—Referred to the recurrence of decay in cavities where a thin layer of partly decalcified dentine, supposed to be disinfected, had been left. When this had been covered by a cap of oxy-chloride, he was of the opinion it acted as an antiseptic.

*Dr. Siddall*—Recommended the use of the cements under large gold fillings.

*Dr. J. M. Whitney*, of Honolulu—Said that he had been on the Sandwich Islands for fourteen years, and during a part of that time he was the only dentist there. He had tried to keep pace with the advance of thought, but had never discovered anything that would act as a substitute for gold in the filling of teeth. He gave a very interesting description of the dental diseases prevalent upon the islands.

*Dr. W. P. Horton*—Said that there was a continued growth of dental decay, and an increase of dental diseases, and evidently this was coincident with the advance of civilization.

Upon the opening of the morning session of the second day, the subject of Dental Education was presented. The President brought up the question of recommending indigent students to certain eastern colleges as objects for a reduction in the fees demanded, and said that he had been greatly annoyed by such applications. He desired instruction as to the advisability of such certification by the President of the society.

*Dr. J. Taft*—Presented the following:

*Resolved,* That this society hereafter declines to certify any student for a beneficiary scholarship in any college.

After some discussion the resolution was unanimously adopted.

*Dr. J. G. Templeton*—Presented a paper upon "The Effect of Zymotic Diseases on the Dental Organs." He urged that the effects of these diseases were hereditary, and were transmitted from parents to children, and that small-pox, scarlet fever, etc., affected the growth of the permanent teeth in children.

*Dr. H. H. Harrison*—Read a paper upon "Dental Education," that subject being again called up. He said that the question of how the coming dentist should be educated was of paramount importance, because upon it rested the answer of what the profession was to be in the future. To succeed in any profession the student must be possessed of a reasonable preliminary education, must have quick perceptions, gentlemanly instincts, and mechanical ability. The latter is essential, because whatever there is that separates dentistry from general medicine requires a mechanic and an artisan to properly perform.

The necessary mechanical training demands that the student shall have a good preceptor. After he shall have spent two years in careful preliminary study he is prepared to comprehend and appreciate college lectures, to listen to the various theories advanced, and to choose the method of practice that is best adapted to his needs.

*Dr. A. Berry*—Also presented a brief paper upon the same subject. In the discussion that ensued the general tone was that a higher standard of qualification in dental students was necessary, and it was believed that recent agitation of the subject had resulted in a better class of matriculants in dental colleges.

During the afternoon session the Committee on Ethics presented charges against two dentists, who had violated the code of ethics by advertising a noted quack anæsthetic called Vitalized Air.

A veterinary dentist, who had given an equine clinic before the members, was given the privileges of the floor and invited to address the society upon the methods of operating upon the teeth of horses.

On motion, the fourth subject of the programme was taken up, "Tin Foil as a Filling."

*Dr. J. W. Lyder*, the President—Opened the discussion. He recommended that a layer of fibrous or felted tin be folded within a sheet of gold, and both be consolidated together.

*Dr. C. R. Butler*—Exhibited some instruments specially adapted for the manipulation of fibrous tin.

He spoke of the difficulty of making good fillings with this material, but said that with proper instruments and knowledge of how to operate, the most satisfactory results could be obtained. He also spoke of the great improvement in the preparation of the foil. He had not used it folded with gold, as the President had described, but had frequently used the tin first, and placed gold on top. This made a good, durable filling, which he believed better than either all gold or all tin. He described an adhesive material which he used in his operations, for supporting the rubber dam when working on a cuspid. His practice was to insert the tooth in the usual manner through the rubber, and then place the substance around the base of the tooth, when it would adhere and hold the rubber in place without the pain and trouble of clamps or ligatures. The formula for making the substance is as follows: Gum damar, 7 ounces; pure white wax, 4 ounces; Canada balsam, 1 drachm. Melt the gum damar, and add in turn the wax and balsam. Pour on a wet surface, and afterward make into sticks. This substance will adhere to a dry surface.*

*Dr. J. G. Templeton*—Said that he used tin in preference to amalgam. There was a preservative quality in tin that was most gratifying to the practitioner.

At the evening session, Dr. J. H. Siddall read a paper on "Old Engines and New." In connection with it he exhibited a dental engine invented by himself.

The election of officers resulted as follows:

President, Dr. A. Berry, Cincinnati; Vice-President, Dr. C. H. James, Cincinnati; Secretary, Dr. J. H. Warner, Columbus; Treasurer, Dr. Geo. W. Keeley, Oxford. Drs. J. Taft and F. H. Rehwinkle were re-elected members of the State Board of Examiners.

At the morning session of the third day, Dr. W. P. Horton, of the Committee on Amendment of the Dental Law, made the following report:

* This preparation is also very useful in stopping holes accidentally made in the rubber dam.—Editor.

Your Committee on Amendment to the Dental Law would report to your honorable body, and recommend that the portion of the present law giving the appointing power of the Board to the Ohio State Dental Society be retained, and that the number of examiners be left to the discretion of the society; that the law be so amended as to require a registration of all dentists in the State with the Clerk of Court of Common Pleas in each county, and that a certified copy of said registration be sent by said clerk to the chairman of said Board of Examiners; that the penalties for violation of the law be retained about the same as at present.

It was unanimously adopted.

The Committee upon Amendments to the Code of Dental Ethics made their report, and this was followed by a scene which was exceedingly painful to every loyal member of the society. The President refused to recognize Dr. R. G. Warner, one of the members against whom charges had been preferred, but he persisted in being heard, and there ensued a contest of lungs against mallet in which neither was entirely victorious. Finally Dr. Harroun, from the Committee on Ethics, submitted the following report:

Having examined the charges of unprofessional conduct in advertising, preferred against Drs. Warner and Gares, of this city, we find them to be the authors of a card which we deem a violation of the code of ethics of this society, and we hereby recommend that they be expelled or suspended from membership of this society, unless they make acknowledgement to the same.

Upon motion, the report was accepted and the members were declared suspended until they shall make proper acknowledgements.

The Executive Committee reported the following subjects for discussion at the next session, to be held in Columbus, on the last Wednesday in October, 1884:

1. Improvements in artificial dentures.

2. Affections and treatment of the active organs of mastication.

3. Therapeutics in dental practice.

4. The difficulties in filling teeth.

5. Pathological conditions of the mucous membranes of the oral cavity; causes and appropriate treatment.

6. Food, digestion and nutrition, in relation to the healthful condition of the teeth and fluids of the mouth.

7. Histology of dental tissues, as shown in the process of development.

*Dr. F. H. Rehwinkle*—Secretary of the State Board of Dental Examiners, reported that three candidates had presented themselves for examination. Two of them failed in their examination last year, and were now re-examined, and granted certificates of qualification. The third had, after studying the questions submitted to him, asked permission to withdraw, without prejudice to his future appearance.

The society then adjourned.

